# Synergistic Effect of Nano-Silica and Intumescent Flame Retardant on the Fire Reaction Properties of Polypropylene Composites

**DOI:** 10.3390/ma16134759

**Published:** 2023-06-30

**Authors:** Yongliang Wang, Baoqiang Liu, Ruiyang Chen, Yunfei Wang, Zhidong Han, Chunfeng Wang, Ling Weng

**Affiliations:** 1 College of Materials Science and Chemical Engineering, Harbin University of Science and Technology, Harbin 150040, China; 2120210127@stu.hrbust.edu.cn (B.L.); clcxds@163.com (R.C.); xiaochangjunshiwyf@163.com (Y.W.); zhidong.han@hrbust.edu.cn (Z.H.); chunfeng.wang@hrbust.edu.cn (C.W.); l.weng@hrbust.edu.cn (L.W.); 2 Key Laboratory of Engineering Dielectrics and Its Application, Ministry of Education, Harbin University of Science and Technology, Harbin 150040, China

**Keywords:** silica nanoparticles, intumescent flame retardant, polypropylene, charred residues, cone calorimeter

## Abstract

Silica nanoparticles (nano-silica) were used as synergistic agents with ammonium polyphosphate (APP) and pentaerythritol (PER) to enhance flame retardancy of polypropylene (PP) in this research. The composites were prepared using a melt-mixing method. The influences of nano-silica on the fire performance of composites were thoroughly discussed, which promotes understanding of nano-silica on the flame-retardant performance of polypropylene composite. Scanning electron microscope (SEM) and energy-dispersive spectrometer (EDS) results indicated that the nano-silica with a diameter of about 95 ± 3.9 nm were dispersed favorably in the composite matrix, which might elevate its synergistic effect with intumescent flame retardant and improve the flame retardancy of polypropylene composite. The synergistic effects between nano-silica and intumescent flame retardant on PP composites were studied using the limiting oxygen index (LOI), UL-94 test, and cone calorimeter test (CCT). The total amount of flame retardant was maintained at 30%. When the dosage of nano-silica was 1 wt.%, the LOI value of PP/IFR/Si1.0 composite reached 27.3% and its UL-94 classification reached V-1. Based on the parameters of the CCT, the introduction of nano-silica induced composites with depressed heat release rate (HRR) and peak heat release rate (PHRR). The PHRR of PP/IFR/Si0.5 was only 295.8 kW/m^2^, which was 17% lower than that of PP/IFR. Moreover, the time to PHRR of PP/IFR/Si0.5 was delayed to 396 s, which was about 36 s later than that without nano-silica. EDS was used to quantitatively analyze the distribution of silica in charred residue. The EDS results indicated that the silica tended to accumulate on the surface during the fire. The surface accumulation characteristic of silica endows it with the enhanced flame-retardant properties of polypropylene composite at a very small dosage (as low as 1 wt.%).

## 1. Introduction

Polypropylene, as one of the commodity polymers, is a low-cost thermoplastic with excellent chemical resistance, low dielectric constant (about 2.2~2.4), and low density (only 0.90~0.91 g/cm^3^, about 60% of polyvinyl chloride) [1,2,3]. The combination of these features with ease of processing has led to a wide range of commercial uses of polypropylene. However, the flammability characteristics of polypropylene (limiting oxygen index value is only 17.5%) limit its application [4,5].

Traditionally, bromine-containing compounds are the most effective and economic flame retardants of polypropylene. Wilkie et al., reported that the addition of bromine-containing compounds can enhance the fire retardancy of polystyrene and polypropylene nanocomposites at very low loading (approximately 6% or less) and a V-0 rating in the UL-94 protocol was obtained [6]. Although bromine-containing polymer composites show low acute toxicity, the composites would produce environmental problems during thermal degradation, such as the release of toxic products. Thus, the uses of bromine-containing flame retardants have been limited or banned in more and more regions around the world. As one of the important halogen-free candidates, intumescent flame retardant (IFR) has been widely used to enhance the flame retardancy of polypropylene composites. Thanh et al., synthesized an efficient intumescent flame retardant via microencapsulation of ammonium polyphosphate using a bioepoxy resin shell [7]. When the microencapsulated form of ammonium polyphosphate was introduced to the polypropylene matrix, the heat release rate of the matrix decreased by 35% and an increased amount of charred residue was obtained during cone calorimeter tests. However, traditional intumescent flame retardants have some shortcomings in flame retardancy of polypropylene, such as low efficiency and poor water resistance [8,9,10]. The low efficiency of intumescent flame retardant induced that a relatively high dosage of IFR (about 25~35%) is needed for expected flame retardant properties, which in turn deteriorated the mechanical and electrical properties of polypropylene composites [11].

To enhance the efficiency of intumescent flame retardant, a series of strategies have been reported, such as microencapsulation to improve its water resistance [12], novel char-forming agents [13], and synergistic agents as additives [10,14,15,16]. It has been proven that synergistic agents (such as nanocaly [17], zeolites [18], metal–organic frameworks [19], graphene [20], zinc borate, and fume silica [21,22]) can effectively improve flame retardancy of polypropylene composites at a relatively low dosage (commonly less than 3 wt.%). Bayramli et al., reported that boron compounds show a maximum flame retardancy effect at 3 wt.% loading and the zinc borate-containing PP composites show the highest rating (V0) using the UL-94 test [23]. However, when the amount of zinc borate exceeds 3 wt.%, the samples show decreased charred residue and a second peak of heat release during the cone calorimeter test.

In the past decades, the application of nano-silica as a synergistic agent in the field of flame retardants has drawn more and more attention, which is due to its accumulation in the vicinity of the composite surface during fire, low thermal conductivity, and low cost. Ye et al., reported the synergistic effect of fumed silica on intumescent flame-retardant polypropylene [24]. They found that a suitable amount of fumed silica can greatly reduce heat release and mass loss rate. However, the synergistic mechanism of fumed silica on intumescent flame-retardant polypropylene was not involved. Moreover, many researchers have reported that aggregation of nano-silica or fumed silica in the polymeric matrix was unavoidable, which limits its application [25]. Moreover, the flame-retardant mechanism of nano-silica with IFR is far from being understood.

To clarify the synergistic effects between nano-silica and intumescent flame retardant on the fire performance of polypropylene composites, nano-silica with a diameter about 95 ± 3.9 nm were synthesized using a two-step method and polypropylene composites with well-dispersed nano-silica in the matrix were obtained using a melt-mixing method. The synergistic effect of nano-silica with ammonium polyphosphate (APP) and pentaerythritol (PER) on fire reaction properties of polypropylene composites was exhaustively discussed, and the mechanism of synergistic effects relied on the surface accumulation characteristic of silica.

## 2. Materials and Methods

### 2.1. Materials

Commercial polypropylene (PP, Q/SY 9B, melt flow index 22 g/10 min) was supplied by Sinopec Yangzi Petrochemical Co., Ltd., Nanjing, China. Ammonium polyphosphate ((NH_4_PO_3_)_n_, APP) and pentaerythritol (C(CH_2_OH)_4_, PER) were supplied by Macklin Chemical Reagent Co., Ltd., Shanghai, China. Cetyltrimethylammonium bromide (CTAB) and tetraethyl orthosilicate (TEOS) were bought from Sinopharm Chemical Reagent Co., Ltd., Shanghai, China. All chemicals were used without further purification.

### 2.2. Synthesis of Silica Nanoparticles

Silica nanoparticles (nano-silica) were synthesized using a two-step method [26]. Firstly, 1 g of CTAB was dissolved in an ammonium hydroxide solution and the pH value of the mixture was adjusted to 11. Secondly, 10 mL of diluted TEOS solution (0.2 M in ethanol) was added dropwise into the above mixture under vigorous stirring, and the stirring was continued for 3 h. After that, 5 mL of TEOS solution (1.0 M in ethanol) was added and the suspension was vigorously stirred for 1 h. Then, the suspension was aged at 50 °C for 24 h. The white precipitate was collected using centrifugation at rpm for 10 min and washed with deionized water and ethanol three times. Finally, the nano-silica was obtained after the samples were freezing dried and calcinated at 400 °C.

### 2.3. Preparation of Intumescent Flame Retardant Polypropylene Composites

PP, APP, PER, and nano-silica were dried in an oven at 80 °C overnight before use. All the polypropylene composites were prepared using a melt-mixing method. Firstly, APP, PER, and nano-silica were premixed in a plastic cup. Then all the raw materials were added to a torque rheometer (RM-200A, Harp Electrical Technology Co., Ltd., Harbin, China). The temperature and roller speed of the torque rheometer were maintained at 180 °C and 60 rpm, respectively, for 8 min. Finally, the melt-blended composites were molded into sheets (10 mm × 10 mm × 3 mm) and intumescent flame-retardant polypropylene composites were obtained. Detailed formulations of the intumescent flame-retardant polypropylene composites are listed in Table 1. The total amount of flame retardant is maintained at 30%. Based on our previous work, the optimum ratio of APP and PER was 2:1, and the dosages of nano-silica were kept at 0.5, 1.0, 1.5, and 2.0 wt.%.

### 2.4. Characterization

The limiting oxygen index (LOI) test was measured using an oxygen index meter (HC-2, Jiangning Analysis Instrument Company, Nanjing, China) according to the ASTM D2863-97 standard [27]. The dimensions of samples were 100 mm × 6.7 mm × 3 mm. UL-94 vertical burning test was carried out using a CZF-III horizontal and vertical burning tester (Jiangning Analysis Instrument Company) according to the ASTM D3801 standard [28]. Dimensions of the samples for the UL-94 vertical burning test were 127 mm × 12.7 mm × 3 mm. To evaluate the fire behavior of polypropylene composites, the samples were tested using an FTT cone calorimeter (Fire Testing Technology, East Grinstead, UK) under the ISO 5660 standard [29]. The thickness of all samples for the cone calorimeter test was 3 mm and the incident heat flux from the cone heater was 35 kW/m^2^. The morphologies of charred residues after cone calorimeter test were investigated using an FEI Apreo scanning electron microscope (SEM) at an acceleration voltage of 5 kV. Moreover, energy-dispersive spectrometry (EDS) was used for the elemental composition and elemental distribution of charred residues.

## 3. Results and Discussion

### 3.1. Effect of Nano-Silica on the LOI Values and UL-94 Ratings of Polypropylene Composites

The polypropylene composites’ reactions to small flame were tested using LOI and UL-94 vertical burning tests. Table 2 lists the LOI values and UL-94 ratings of polypropylene composites with different dosages of nano-silica.

The LOI value of neat PP is only 17.5%, which means it is highly combustible, and it cannot pass the UL-94 vertical burning tests. When 30 wt.% IFR was introduced, the LOI value of PP/IFR composite was significantly improved to 25.4%, but it did not pass the UL-94 tests. When the addition of nano-silica was 0.5 wt.%, the LOI value of PP/IFR/Si0.5 composite reached 26.7%. It is worth noting that UL-94 classification of PP/IFR/Si0.5 composite reached V-2 rating, which means nano-silica can effectively improve the flame retardancy of intumescent flame-retardant polypropylene even at a very small dosage. When the dosage of nano-silica was increased to 1 wt.%, the PP/IFR/Si1.0 composite showed increased LOI value, and its UL-94 classification reached V-1. However, with the dosages of nano-silica further increased to 1.5 wt.% and 2 wt.%, the flame retardancy of polypropylene composites deteriorated. The LOI values of PP/IFR/Si2.0 decreased to 26.4%, which are still higher than that of PP/IFR. Similar results have been reported in the literature and our previous report [30]. Qu et al., reported that when 2 wt.% fumed silica was added to polystyrene composites, its LOI value was about 5 units lower than that of polystyrene composites without fumed silica [24]. Excessive addition of nano-silica might lead to high melt viscosity of composite during fire, which in turn suppresses the effectiveness of intumescent flame retardants. Taking the LOI value into consideration, the effect of synthesized nano-silica on the flame retardancy of polypropylene composites is better than that of fumed silica in the literature, which was might due to the fine distribution of synthesized nano-silica in composites. The distribution of nano-silica in composites is discussed in the following parts.

### 3.2. Morphologies of Nano-Silica and Its Dispersion in Polypropylene Composites

**Figure 1:**
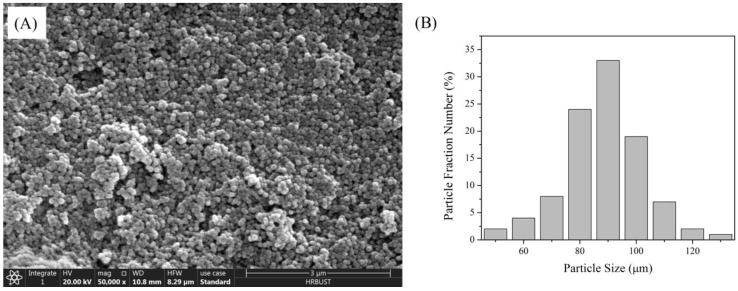
SEM micrograph (**A**) and size distribution (**B**) of synthesized nano-silica.

Figure 1A shows the SEM morphology of the synthesized nano-silica. Spherical nano-silica with uniform particle size distribution can be observed. Image Pro Plus was used to analyze Figure 1A. After automatic recognition of the image and manual particle segmentation, the results from Image Pro Plus indicated that the average diameter of nano-silica was about 95 ± 3.9 nm (statistical result illustrated in Figure 1B).

**Figure 2:**
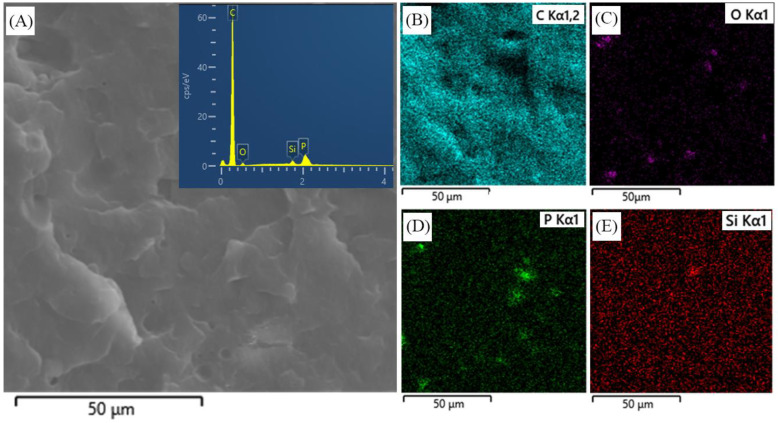
Morphology and elemental mapping of polypropylene composite PP/IFR/Si2. SEM morphology of composites (**A**), elemental carbon (**B**), elemental oxygen (**C**), elemental phosphorus (**D**), and elemental silicon (**E**).

The dispersion of intumescent flame retardant and nano-silica was characterized using SEM and EDS. The morphology of the composite and the entire image elemental mapping of C, P, O, and Si are shown in Figure 2. The bright dots represent C (blue), O (pink), P (green), or Si (red). Figure 2B–D revealed that the inorganic flame retardants (APP and PER) were dispersed favorably in the composite matrix. No significant interface was shown between polypropylene and flame retardant, and slight aggregation of phosphorus and oxygen were shown. Figure 2E confirms that the nano-silicon is homogenously distributed in the composites. 

Table 3 shows the mechanical properties of polypropylene composite with different dosages of nano-silica. With a nano-silica loading increase from 0 to 2 wt.%, the tensile strength of the composites slightly increased from 16.35 ± 0.24 MPa to 18.99 ± 0.41 MPa. Moreover, the elongation at the break of composites shows a similar trend. The tensile strength increase in polypropylene composite was induced due to two reasons. Firstly, the total amount of flame retardant was maintained at 30%, and the nano-sized silica had a positive effect on mechanical properties compared to APP and PER. On the other hand, nano-silicon was homogenously distributed in the composites (Figure 2E), which endowed the composites with enhanced mechanical performance.

### 3.3. Heat Release Properties of Polypropylene Composites

Cone calorimetry based on the oxygen consumption principle has been proved an effective method for predicting the combustion behaviors of materials on a bench scale. Here, several important parameters from cone calorimeter tests, such as peak heat release rate (PHRR), heat release rate (HRR), total heat release (THR), total smoke release (TSR), mass loss rate (MLR), and specific extinction area (SEA) were used to quantitatively evaluate the fire behavior of polypropylene composites.

**Figure 3:**
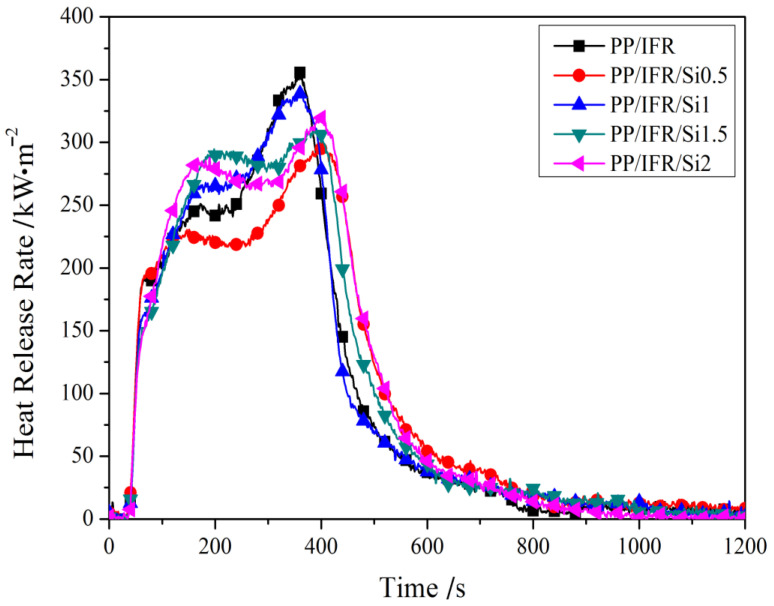
Effect of nano-silica on the heat release rate of polypropylene composites at a 35 kW/m^2^ heat flux.

HRR curves of polypropylene composites with different dosages of nano-silica at a 35 kW/m^2^ heat flux are presented in Figure 3. HRR is considered an important parameter for reflecting fire spread. As can be seen from Figure 3, all the samples show similar heat release within 100 s after ignition. Moreover, all HRR curves show two peak characteristics. The first peak corresponded to the formation of an intumescent shielding layer and the second heat release peak was attributed to the out-of-protection of the shielding layer. For PP/IFR composite, the highest peak heat release rate was 355.5 kW/m^2^ at around 360 s. When 0.5 wt.% nano-silica was introduced, the PP/IFR/Si0.5 sample showed depressed HRR curve peak heat release. The PHRR of PP/IFR/Si0.5 was only 295.8 kW/m^2^, which was 17% lower than that of PP/IFR. Moreover, the time of PHRR of PP/IFR/Si0.5 was delayed to 396 s, which was about 36 s later than that without nano-silica.

A comparison of HRR curves between PP/IFR and PP/IFR/Si0.5 indicated that 0.5 wt.% nano-silica enhanced the effectiveness of the charred layer, which in turn induced a reduction in peak heat release of 17%. However, with the nano-silica dosage increase, the PHRR values of PS/IFR/Si1, PS/IFR/Si1.5, and PS/IFR/Si2 increased to 339.7, 307.7, and 320.9 kW/m^2^, respectively, which are still lower than composite without nano-silica.

**Figure 4:**
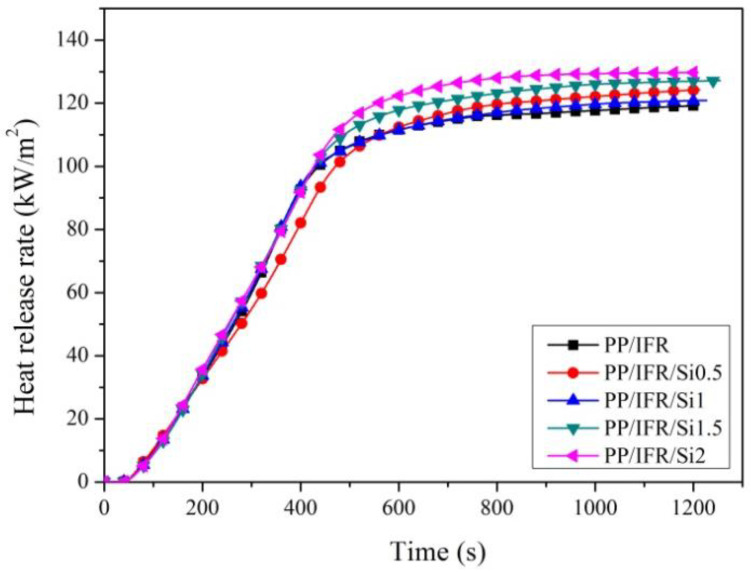
The influence of nano-silica on the total heat release of intumescent flame-retardant polypropylene composites.

Figure 4 presents the total heat release (THR) curves versus time for the polypropylene composites. Compared to the PP/IFR composite, the THR of PS/IFR/Si0.5 was only 124.2 MJ/m^2^, which is lower than that of PP/IFR. Notably, when the dosage of nano-silica increased to 1.0 wt.%, the THR of PS/IFR/Si1.0 further decreased to 120.8 MJ/m^2^. The decrease in total heat release indicates a decrease in polymer matrix degradation and an increase in charred residual, which proves that the addition of 1.0 wt.% nano-silica is beneficial for reducing the decomposition of the polypropylene matrix. Table 4 lists the cone calorimeter test data of polypropylene-based samples with different nano-silica dosages, which provides positive evidence that nano-silica and intumescent flame retardant have a synergistic effect at specific addition amounts of nano-silica. However, the THRs of PS/IFR/Si1.5 and PS/IFR/Si2 were higher than that of PS/IFR/Si1.0, which is consistent with the HRR curves in Figure 3.

### 3.4. Smoke Property

Smoke is an important parameter for evaluating the flame retardant property of materials. The smoke character not only reflects the combustion of materials during a fire but also impacts human survivability during a fire. The smoke properties of polypropylene composites were discussed based on the parameters of smoke release rate (SRR), total smoke release (TSR), and specific extinction area (SEA).

**Figure 5:**
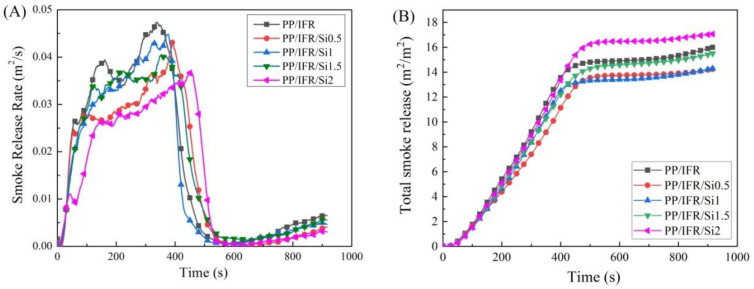
Effect of nano-silica on smoke release properties of polypropylene composites. (**A**) smoke release rate; (**B**) total smoke release.

The smoke release rate (SRR) curves and total smoke release (TSR) curves of polypropylene composites are shown in Figure 5. From Figure 5A, it can be seen that the SRR curve of PP/IFR expressed a sharp two-peak characteristic and its peak smoke release rates were 0.039 m^2^/s and 0.047 m^2^/s, respectively. When 0.5 wt.% nano-silica was added, the first peak smoke release rate decreased to 0.027 m^2^/s (about 31% lower than that of PP/IFR), which evidenced a significant smoke suppression property of nano-silica. With the dosage of nano-silica increased to 1.0 wt.%, a slower smoke release rate and a decreased second peak smoke release rate (0.036 m^2^/s, about 23.4% lower than that of PP/IFR) can be seen. However, the smoke suppression performances of PP/IFR/Si1.5 and PP/IFR/Si2 were deteriorated than that of PP/IFR/Si1. A similar trend was shown for TSR curves in Figure 5B. The TSR value of PP/IFR was 1689.1 m^2^/m^2^. The TSR values of PP/IFR/Si0.5 and PP/IFR/Si01.0 decreased to 1509.6 m^2^/m^2^ and 1508.8 m^2^/m^2^, respectively, which were about 11% lower than that of PP/IFR.

Specific extinction area (SEA) is a parameter based on the release of smoke per unit time during sample combustion. The SEA values for polypropylene composites were listed in Table 4. The SEA value of polypropylene composite without nano-silica (PP/IFR) is 507.8 m^2^·kg^−1^. With the addition of nano-silica, the SEA values of PP/IFR/Si0.5 and PP/IFR/Si1 decreased by 23.5%, 17.1%, and 22.7% compared to that of PP/IFR, respectively. However, when the dosages of nano-silica reached 1.5 wt.% and 2.0 wt.%, the PP/IFR/Si1.5 and PP/IFR/Si2 composites showed enhanced SEA values. In terms of TSR values and SEA values, the nano-silica has a positive influence on the suppression of smoke release at a dosage lower than 1 wt.%.

### 3.5. Flame Retardancy Performance

**Figure 6:**
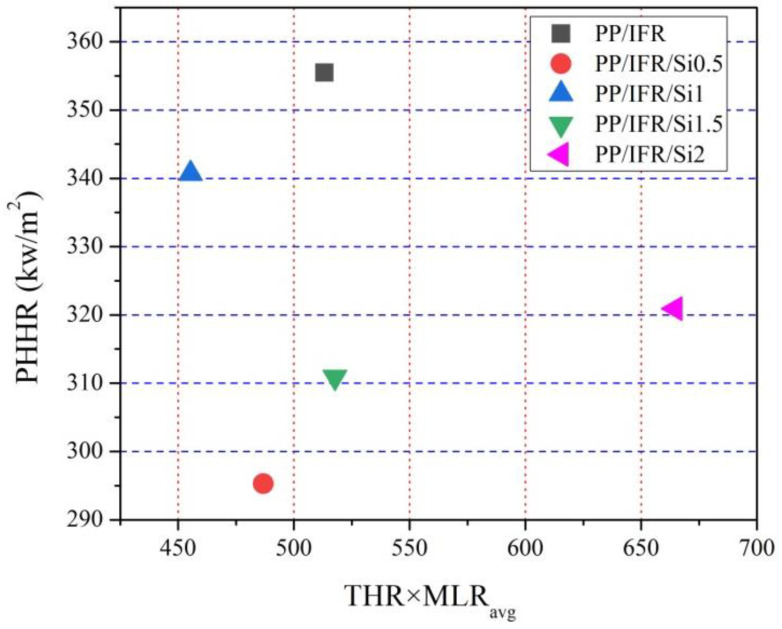
PHRR with respect to THR×MLR_avg_ for fire performance evaluation of polypropylene composites using nano-silica as the synergistic agent.

A single parameter of the cone calorimeter test, such as HRR and THR, is not sufficient for evaluating the fire performance of polypropylene composites [30]. These parameters reflect different characteristics of materials in the fire. The derived parameters based on a cone calorimeter test can provide a comprehensive evaluation of its flame-retardant performance. Here, the plot of PHHR as a function of THR×MLR_avg_ was used to evaluate the flame-retardant performance of polypropylene composites at different dosages of nano-silica. As is known, PHHR can reflect the fire spread rate of samples and THR×MLR_avg_ can reflect the degradation character of material during the fire. Based on the results from Figure 6, PP/IFR/Si0.5 and PP/IFR/Si1 have enhanced fire retardancy performance and PP/IFR/Si2 shows deteriorative fire retardancy performance. These evaluation results are consistent with the heat release results and smoke release results.

### 3.6. Analysis of Charred Residue

Based on the results above, the flame retardancy and smoke suppression of the polypropylene composites were improved significantly by the addition of nano-silica less than 1 wt.%. To gain insight into the mechanism of nano-silica in relation to the flame behavior of polypropylene composites, it is necessary to investigate the morphologies and compositions of charred residue. Here, scanning electron microscope (SEM) and energy-dispersive spectrometer (EDS) were used to analyze the charred residue of samples after the cone calorimeter test.

**Figure 7:**
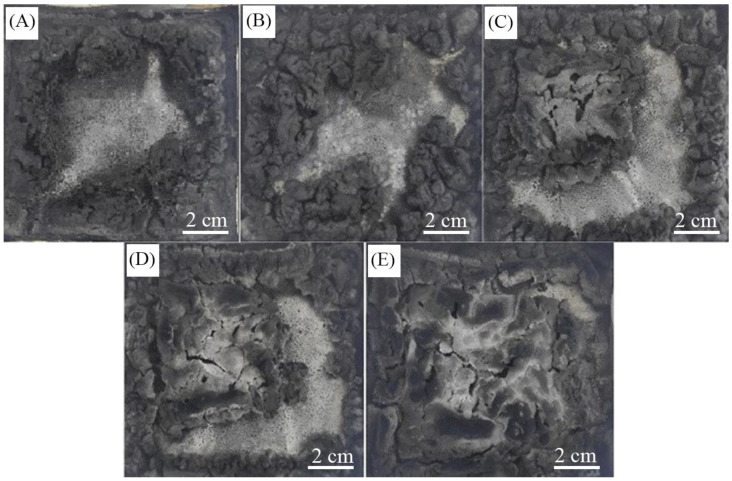
Morphologies of charred residues of polypropylene composites after cone calorimeter test. (**A**) PP/IFR; (**B**) PP/IFR/Si0.5; (**C**) PP/IFR/Si1; (**D**) PP/IFR/Si1.5; (**E**) PP/IFR/Si2.

The digital morphologies of charred residues after the cone calorimeter test are shown in Figure 7. From Figure 7, it can be easily concluded that nano-silica has a significant positive influence on the formation of charred residues. The charred residue of the polypropylene composites without nano-silica shown in Figure 7A was loose, with holes and cracks. With the dosages of nano-silica increased from 0.5 to 2.0 wt.%, the compactness and continuity of the charred residues were enhanced, especially for Figure 7D and Figure 7E. The morphologies of charred residue imply that the synergistic effect between nano-silica and intumescent flame retardant occurs in the condensed phase. With the compactness and continuity enhancement, the charred layer can prevent or delay heat and gas transfer between the polymer matrix and surface, which in turn reduces the heat release and smoke release.

**Figure 8:**
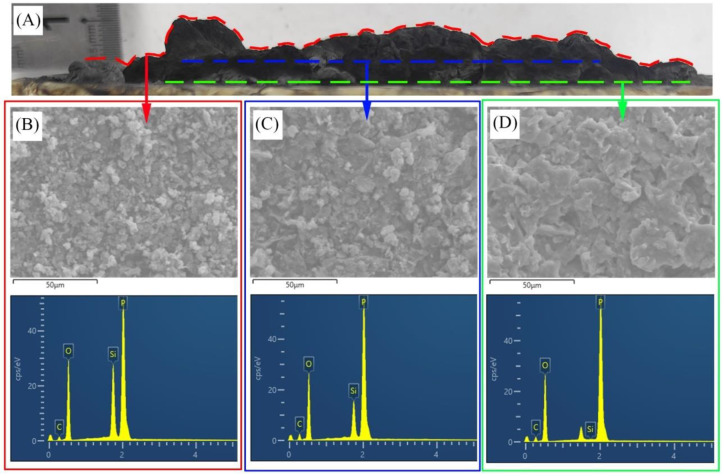
SEM micrographs (**A**) and EDS patterns of the surface (**B**), middle (**C**), and bottom (**D**) parts of the residue of PP/IFR/Si1.

To clarify the influence of nano-silica on the condensed phase during the fire, EDS was used to quantitatively analyze the distribution of silica in charred residue. The side-view morphology of the charred residue of PP/IFR/Si1 is shown in Figure 8A. The SEM micrographs and EDS patterns of the surface, middle, and bottom parts of the char layer are illustrated in Figure 8B–D, respectively. Moreover, the EDS results are listed in Table 5. It can be concluded from Figure 8 that the charred layer of PP/IFR/Si1 shows a compact structure from surface to bottom. The compact charred layer was due to the synergistic effect of the nano-silica and intumescent flame retardant. It is worth noting that the distribution of silicon from the surface to the bottom of the charred layer is nonuniform. As listed in Table 5, the silicon content at the surface of the charred layer was as high as 8.02 wt.%, but its content decreased to 0.06 wt.% at the bottom of the charred layer. The EDS results indicated that the silica tended to accumulate on the surface during the fire. The surface accumulation characteristic of silica endows it with the enhanced the flame-retardant properties of polypropylene composite at a very small dosage (as low as 1 wt.%).

## 4. Conclusions

Spherical silica nanoparticles (nano-silica) with uniform particle size about 95 ± 3.9 nm were synthesized and used as the synergist for intumescent flame-retardant polypropylene composites. Based on the parameters from a CCT, the introduction of nano-silica induced composites with depressed heat release rate (HRR) and peak heat release rate (PHRR). The plot of PHHR with respect to THR×MLR_avg_ indicated that the PP/IFR/Si0.5 and PP/IFR/Si1 show enhanced fire retardancy performance. The PHRR of PP/IFR/Si0.5 was only 295.8 kW/m^2^, which was 17% lower than that of the composite without nano-silica. To understand the synergistic mechanism between nano-silica and intumescent flame retardant, an energy-dispersive spectrometer (EDS) and element mapping were used to investigate the composition and elemental distribution of the charred residue of samples after cone calorimetry. The EDS results indicated that the silica tended to accumulate on the surface during the fire. The silicon content at the surface of the charred layer was as high as 8.02 wt.%, but its content decreased to 0.06 wt.% at the bottom of the charred layer. The surface accumulation characteristic of silica endows it with the enhanced flame-retardant properties of polypropylene composite at a very small dosage (as low as 1 wt.%). The trend might apply to other polymer matrixes, and combining nano-silica with other newly developed high-efficiency intumescent flame retardants might induce surprising performance.

## Figures and Tables

**Table 1:** Components and description of intumescent flame-retardant polypropylene composites.

Samples	Description	PP (wt.%)	APP (wt.%)	PER (wt.%)	Nano-Silica (wt.%)	Stearic Acid (wt.%)	Antioxidants (wt.%)
PP	PP	99	0	0	0	0.5	0.5
PP/IFR	PP/IFR	70	19.35	9.65	0	0.5	0.5
PP/IFR/Si0.5	PP/IFR with 0.5 wt.% nano-silica	70	19	9.5	0.5	0.5	0.5
PP/IFR/Si1	PP/IFR with 1.0 wt.% nano-silica	70	18.6	9.4	1.0	0.5	0.5
PP/IFR/Si1.5	PP/IFR with 1.5 wt.% nano-silica	70	18.33	9.17	1.5	0.5	0.5
PP/IFR/Si2	PP/IFR with 2.0 wt.% nano-silica	70	18	9	2.0	0.5	0.5

**Table 2:** The flammability data of intumescent flame-retardant polypropylene composites.

Samples	UL-94 Rating	LOI (%)
PP	NR	17.5
PP/IFR	NR	25.4
PP/IFR/Si0.5	V-2	26.7
PP/IFR/Si1	V-1	27.3
PP/IFR/Si1.5	NR	27.6
PP/IFR/Si2	NR	26.4

**Table 3:** Mechanical properties of polypropylene composite.

Samples	Tensile Strength (MPa)	Elongation at Break (%)
PP/IFR	16.35 ± 0.24	45 ± 4
PP/IFR/Si0.5	17.07 ± 0.29	47 ± 4
PP/IFR/Si1	17.31 ± 0.34	48 ± 4
PP/IFR/Si1.5	18.03 ± 0.34	48 ± 5
PP/IFR/Si2	18.99 ± 0.41	50 ± 5

**Table 4:** Cone calorimeter data for intumescent flame-retardant polypropylene composites.

Sample	TTI (s)	PHRR (kW·m^−2^)	Time to PHRR (s)	THR (MJ·m^−2^)	MLR_avg_ (g·s^−1^·m^−2^)	SEA (m^2^·kg^−1^)	TSR (m^2^·m^−2^)
PS/IFR	32	355.5	360	126.1	4.07	507.8	1689.1
PS/IFR/Si0.5	30	295.3	396	124.2	3.92	421.2	1509.6
PS/IFR/Si1	31	340.7	362	120.8	3.77	392.5	1508.8
PS/IFR/Si1.5	30	310.9	395	127.2	4.07	441.1	1637.4
PS/IFR/Si2.0	31	320.9	398	129.8	5.12	518.1	1802.5

**Table 5:** Elemental weight fractions of different locations of charred residue of PP/IFR/Si1 using energy-dispersive spectrometry.

Location of Char Residue	C (wt.%)	O (wt.%)	P (wt.%)	Si (wt.%)
Surface	18.76	55.34	17.87	8.02
Middle	26.85	51.84	17.24	4.07
Bottom	31.51	52.09	16.33	0.06

## Data Availability

Not applicable.
